# Influence of age on force and re-lengthening dynamics after tetanic stimulation withdrawal in the tibialis anterior muscle

**DOI:** 10.1007/s00421-023-05198-0

**Published:** 2023-04-18

**Authors:** M. Cogliati, A. Cudicio, M. Benedini, H. V. Cabral, F. Negro, C. Reggiani, C. Orizio

**Affiliations:** 1 Department of Clinical and Experimental Sciences, University of Brescia Viale Europa, 11, 25123 Brescia, Italy; 2 Centre of Research on the Neuromuscular Function and the Adapted Motor Activity, University of Brescia Viale Europa, 11, 25123 Brescia, Italy; 3 Department of Biomedical Sciences, University of Padova, Padova, Italy; 4 Science and Research Center, ZRS, Koper, Slovenia

**Keywords:** Aging, Relaxation process, Rate of torque reduction, Stimulation, MMG

## Abstract

**Purpose:**

During alternate movements across a joint, the changeover from one direction of rotation to the opposite may be influenced by the delay and rate of tension reduction and the compliance to re-lengthening of the previously active muscle group. Given the aging process may affect the above-mentioned factors, this work aimed to compare the dynamics of both the ankle torque decline and muscle re-lengthening, mirrored by mechanomyogram (MMG), in the tibialis anterior because of its important role in gait.

**Methods:**

During the relaxation phase, after a supramaximal 35 Hz stimulation applied at the superficial motor point, in 20 young (Y) and 20 old (O) subjects, the torque (T) and MMG dynamics characteristics were measured.

**Results:**

The T and MMG analysis provided: (I) the beginning of the decay after cessation of stimulation (T: 22.51 ± 5.92 ms [Y] and 51.35 ± 15.21 ms [O]; MMG: 27.38 ± 6.93 ms [Y] and 61.41 ± 18.42 ms [O]); (II) the maximum rate of reduction (T: − 110.4 ± 45.56 Nm/s [Y] and − 52.72 ± 32.12 Nm/s [O]; MMG: − 24.47 ± 10.95 mm/s [Y] and − 13.76 ± 6.54 mm/s [O]); (III) the muscle compliance, measuring the MMG reduction of every 10% reduction of torque (bin 20–10%: 15.69 ± 7.5[Y] and 10.8 ± 3.3 [O]; bin 10–0%: 22.12 ± 10.3 [Y] and 17.58 ± 5.6 [O]).

**Conclusion:**

Muscle relaxation results are different in Y and O and can be monitored by a non-invasive method measuring physiological variables of torque and re-lengthening dynamics at the end of the electromechanical coupling previously induced by the neuromuscular stimulation.

## Introduction

The alternated movement of a joint, such as the ankle, knee and elbow, is evidently related to the coordinated, alternated activation of the flexor and extensor muscles acting across the hinge. While the muscles providing an angular momentum toward the joint rotation direction are referred as agonist, the muscle group generating an opposite angular momentum is defined as antagonist. Every time the joint rotation is reversed, there is a swap between the roles of antagonist and agonist muscles. The changeover from one direction of rotation to the opposite as well as the re-lengthening phase of the past agonist is influenced by: (a) the tension reduction of the previously active muscle group, (b) the compliance of this last to re-elongation by the new active agonist. Consequently, the assessment of biomechanical parameters during these two aforementioned processes could provide functional data to characterize the muscular features affecting the agonist–antagonist sequential activity.

Gait can be considered as a global alternating movement resulting from the combination of several joints alternating flexion–extension sequences. According to Westerblad et al. (1997), “slowed relaxation of antagonist muscle might counteract the desired movement during rapid, alternating movements”. Thus, during normal locomotion, the slowing of relaxation of the previously active muscle group may greatly affect the dynamic of the joint transition from a rotational direction to the following. This may influence the locomotion parameters, particularly in aged subjects. Indeed, data about gait analysis suggest that age influences the gait in length stride and phase duration (Mulas et al. 2021; Fukuchi et al. 2019). Changes in gait in elderly have been also associated with an increased risk of institutionalization and death. For instance, reduction of the walking speed has been demonstrated to be predictive of life expectancy (Studenski et al. 2011). Furthermore, disturbances in balance and gait have been implicated in an increased risk of falls (Osoba et al. 2019).

On this base, it seems important to evaluate the dynamics of muscle tension reduction and re-lengthening after activation in young and old subjects. The functional parameters describing the force and re-lengthening process during the relaxation phase are not comparatively well described in the above-mentioned populations. The force decrement onset delay, from the myoelectric activity cessation, or its velocity of decay in different conditions such as pre- and post-stretching maneuver (Longo et al. 2017, 2014) or before and after fatigue (Cè et al. 2014a, 2014b), has been investigated in the literature. Only few studies report the behavior of tension and surface mechanomyogram (MMG) signal detected by an accelerometer, monitoring the muscle re-elongation, in the relaxation phase when simultaneously recorded (Cè et al. 2014a, 2013b; Esposito et al. 2016; Longo et al. 2014). Indeed, they used the MMG as an indicator of re-lengthening onset, but not of its time dynamics.

To assess the torque and muscle length behavior at the end of muscle contraction, it is possible to use an experimental setup previously described by our group (Cogliati et al. 2020), in which the isometric torque of ankle dorsiflexion and the tibialis anterior length were simultaneously measured by a load cell and surface mechanomyography, respectively. The rationale can be summarized as follows. Since the muscle is a constant volume system, each shortening during a contraction provides an increase in the transverse diameter of the muscle. This dimensional variation can be picked up by a laser sensor. By analogy, during muscle relaxation after activity, the laser distance signal can be considered as an index of the muscle re-elongation process. The study of muscle length changes by surface MMG has already been implemented in detail (Orizio et al. 1996; Yoshitake et al. 2005; Beck et al. 2005). The rationale for adopting MMG as an indirect measure of the muscle length changes, instead of the collection of the ultrasound (US) images from the active muscle, is based on the following considerations: (a) at present the US technique unlikely provides more than 30–60 frames per second with a time resolution of 30–15 ms (too low for a good tracking of the time behavior of re-elongation after cessation of the muscle activity), (b) the experimental setup is quite complex requiring a robotic arm sustaining the US probe immersed in a pool able to accommodate the distal leg, (c) the post-processing of each images to extract the length change is complex and time consuming, (d) the cost of a US system is much greater than a simple laser distance sensor. Finally, the choice of the MMG signal make the replication of the study easy.

An experimental design that can neatly provide basic data about the tension reduction and re-lengthening processes relationship of an active muscle once the activity is withdrawn must be based on stimulated contractions. In this way, it is possible to minimize the uncertainty of the individual fading pattern of the central nervous system drive suspension that may influence the outcome of the changes in the muscle contractile status during the relaxation phase.

Given the possible meaning in determining the mechanical efficiency of alternating movements, the aim of this work was to compare in the tibialis anterior of young and old subjects the dynamics of:

- the torque reduction at the ankle;
- the muscle re-lengthening during the relaxation phase.

It is worth underlining how important the study of tibialis anterior mechanics is, given its major role in the gait cycle for both stabilization of the ankle joint during the early phase of stance and for elevation of the foot during the early phase of swing (Lacquaniti et al. 2012). As a consequence, its relaxation dynamics may deeply influence the timing of transition to the following phases of the gait.

## Materials and methods

### Subjects and measurements

**Fig. 1:**
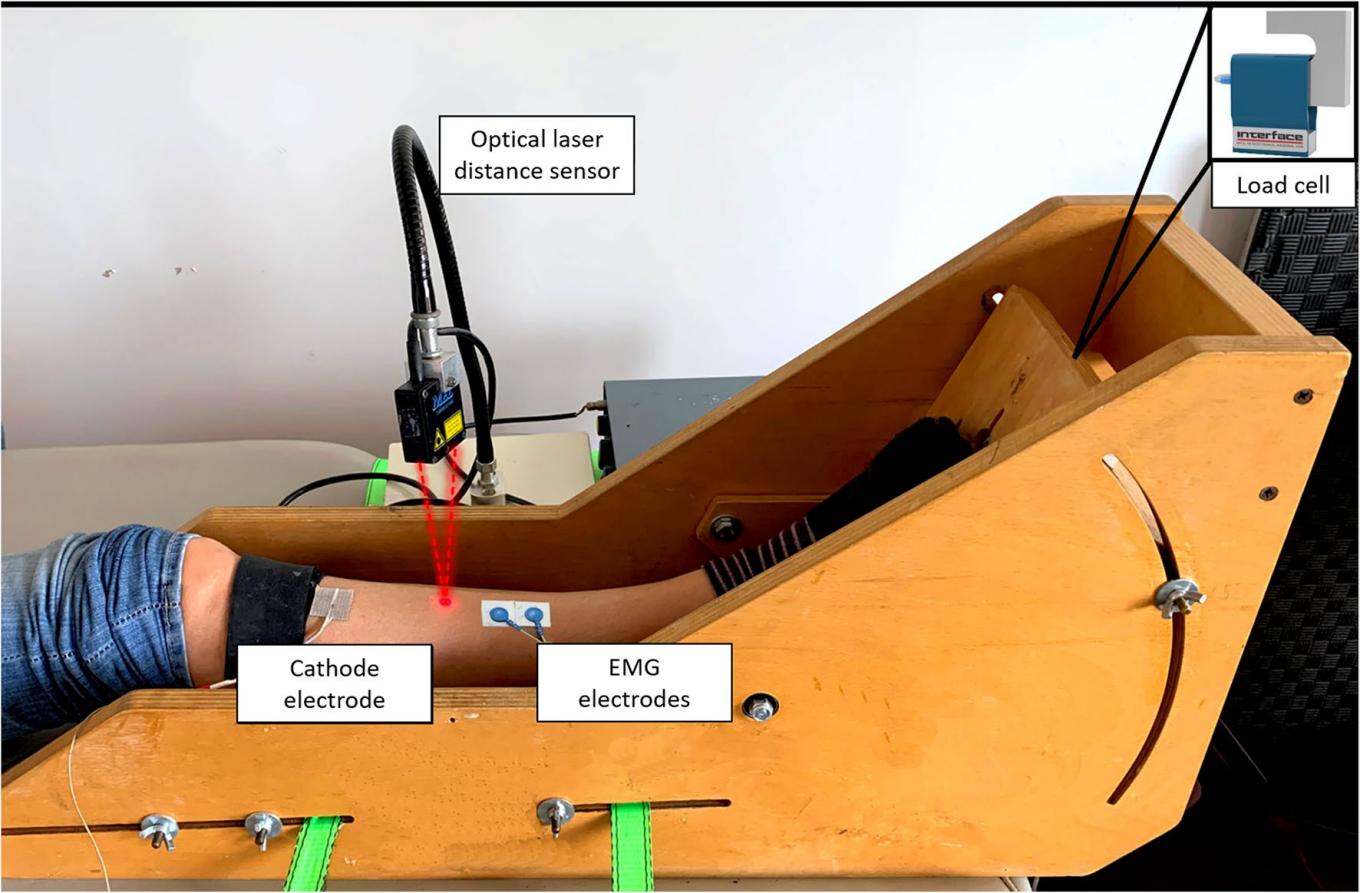
Schematic representation of the experimental setup. The custom-made wooden ergometer for static contraction of the tibialis anterior muscle with the load cell for torque measurement is represented. The cathode at the tibialis anterior main motor point, the optical laser distance sensor pointing at the muscle belly (MMG detection) and two electrodes for the detection of the EMG in differential mode are reported

Twenty recreationally active young participants (10 males and 10 females; age 21–33 years old) and 20 recreationally active older participants (10 males and 10 females; age 65–80 years old) with no orthopedic or neurological disorders were recruited to participate in this study. After receiving a full explanation of the experiments, they provided their written informed consent. The subjects were asked to refrain from caffeine intake and intense physical activity in the 24 h preceding the test. This study was conducted in accordance with the latest version of the Declaration of Helsinki and approved by the local ethical committee. The participants' dominant lower limb was positioned on a specific ergometer equipped with a load cell (Fig. 1), which measured the torque generated during the electrically stimulated contractions of the tibialis anterior muscle (Cogliati et al. 2020). While the hip and the knee were, respectively, fixed at 90° and 180°, the ankle was positioned in a neutral position at 110°. The foot was strapped to the wood plate connected to the load cell (model SM-100 N, by Interface Inc., Scottsdale, US-AZ). The force signal acquired by the load cell was band-pass filtered at 0–64 Hz and amplified (MISO- OT Bioelettronica, Turin, Italy). To get the dorsiflexion torque produced by each subject, the distance between the ankle fulcrum and the load cell at the foot plate was measured and used to convert the force signal in torque [T = F (N) × d (m)]. According to Orizio et al. (Orizio et al. 1999, 2008), the displacement of the tibialis anterior muscle surface was transduced as a mechanomyographic signal using an optical laser distance sensor (M5L/20, MEL Mikroelektronik, Germany). The instrument has the following features: range of measurement ± 10 mm, sensitivity 1 V/mm, linearity 0.6%, resolution < 6 μm, bandwidth 0–10 kHz. The laser beam was pointed to the tibialis anterior muscle belly presenting the largest displacement during the tetanic stimulation. The common position was at about 1 cm from the tibial crest as reported in Fig. 1. The device provided an output DC voltage proportional to the distance between the laser beam head emitter and the reflecting muscle surface. The measure of the distance of the reflecting surface from the laser source was not affected by surface rotation within ± 15° and ± 30° with respect to the short and long axis of the laser head, respectively. The force and MMG were digitized at a frequency of 1024 samples/s (CED-1401 of Cambridge Electronic Design of Cambridge).

An electrical stimulator was used to deliver biphasic rectangular stimuli (100 µs duration of each phase) on the tibialis anterior muscle. The cathode electrode (5 × 5 cm) was placed at the skin region over the main motor point of the tibialis anterior (Fig. 1), which was identified according to Gobbo et al. (2011). The anode electrode (15 × 10 cm) was positioned on the gastrocnemius muscle. By increasing the amplitude of a 1 Hz stimulation train (10 pulses per each 0.1 V amplitude level), from the minimum value of 0.5 V, the maximum stimulation pulse was identified as the stimulus amplitude eliciting the largest single twitch. Three trains of 35 Hz pulses lasting 3 s were administered to the motor point of the muscle with a 1 min pause between stimulations. The surface EMG evoked by the stimulation train was detected by means of two self-adhesive pre-gelled silver electrodes (1 cm in diameter; inter-electrode distance 30 mm). EMG was conditioned using a third-order Butterworth band-pass filter (10–512 Hz). After A/D conversion by CED-1401 (Cambridge Electronic Design, Cambridge, UK), the digitized signals were stored on a PC and sampled at 1024 samples/s.

### Signal processing: analyzed parameters during the relaxation phase

To achieve the purpose of the work, the analysis described here below concerns the relaxation phase of the stimulated tetanic contraction, which has been partly already considered by several studies (Cè et al. 2013b, 2014a, 2013c; Esposito et al. 2016, 2011; Longo et al. 2016).

**Fig. 2:**
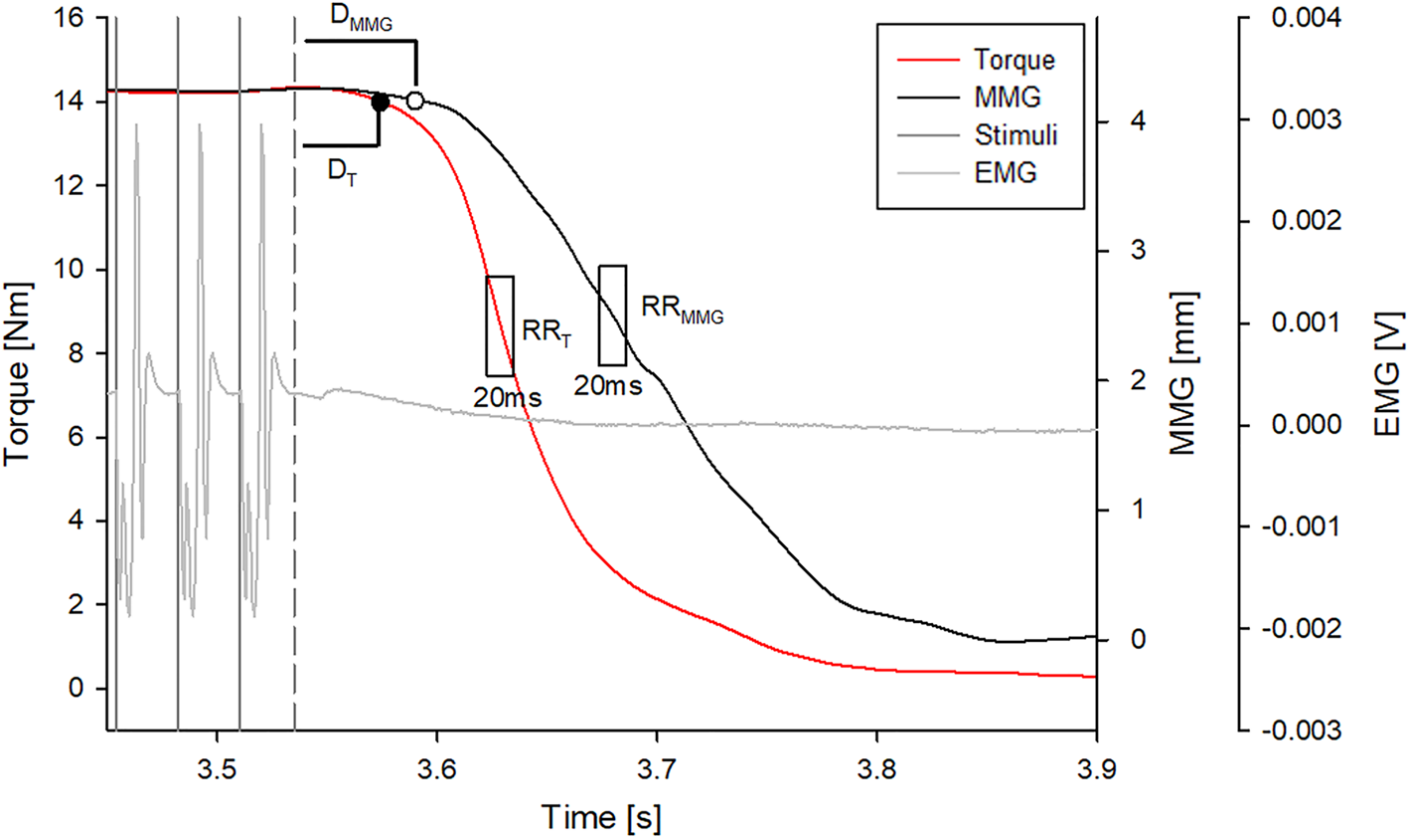
Torque and MMG during the relaxation phase in a representative subject. The 20 ms moving windows on the two signals, used for the maximum rate of reduction identification, are reported. The torque and MMG delays correspond to the time intervals between the end of the evoked EMG activity (dashed gray line) and the beginning of the signal decays (open and filled circles). The EMG signal and the last three stimuli of the 35 Hz train are reported in light and dark gray, respectively

Out of the three stimulation trains, the one with the greatest torque value in the 100 ms time interval before the last stimulus was selected for each subject. The torque and MMG were digitally low-pass filtered at 50 Hz and subsequently normalized to their 100% referred to the average values in 100 ms time interval. The EMG signal was used to identify the end of the electrical activity due to the tetanic stimulation train. The time at which the electrical activity was completed, after the last stimulus, was the time mark at which the evoked EMG reached its average value ± 3 SD calculated from 1 s signal sample before the tetanic stimulation (see Fig. 2).

### Relaxation electromechanical delay

During the relaxation phase, a delay (D) can be observed between the end of electrical activity and the beginning of torque and MMG decay. D was calculated as the time instant when the signals decrease 3 standard deviations of their average value during stimulation, both for torque (D_T_) and MMG (D_MMG_) (Fig. 2).

#### Rate of torque reduction and rate of MMG reduction

The rate of reduction for torque signal (RR_T_) and MMG signal (RR_MMG_) were calculated as the ratio between the Δtorque or ΔMMG and Δtime (Fig. 2). Specifically, a 20 ms moving window with a step of 1 ms was used across the two signals to identify the maximum rate of reduction (Cogliati et al. 2020; Haff et al. 2015). The same calculation was performed on the normalized signals to obtain NRR_T_ and NRR_MMG_.

#### Time interval for 80–20% signal reduction

In addition to the discrete information provided by RR or NRR, the time of reduction of both the normalized torque and MMG (TR_T_ and TR_MMG_) in the range of 80–20% of their reductions was calculated to further characterize the dynamics of signal decay. The selected range allows to compare the time behavior of the two signals from young and old subjects when both are dynamically changing out of the initial and final transients.

#### Muscle compliance

To have a detailed description of the time relationship between the torque decrement and muscle re-lengthening, the amount of relative MMG variation for each of the ten bins of relative torque decrease (from 100 to 0%: 100–90%, 90–80%, 80–70%, …, 10–0%) was calculated. This value mirrors the bin-by-bin muscle compliance (MC) to re-elongation throughout the relaxation process.

#### MMG at the end of torque reduction (MMG_0T_)

The %MMG, amount of re-lengthening left, when torque reduction process was completed and reached 0% was quantified for each subject. The parameter was identified as MMG_0T_ and provides a measure of the whole re-lengthening process efficiency compared to tension reduction: in other words, how much the re-lengthening is incomplete once the force felt is 0.

### Statistical analysis

The data were analyzed using a statistical software (Sigmaplot 11). A two-way analysis of variance (ANOVA) was used to examine the main and interaction effect age (young and old) and signals (torque and MMG) on the D, NRR, and TR. When ANOVA was significant, pairwise comparisons were made with Tukey post hoc test. For muscle compliance, the two factors for ANOVA were age and the relative torque decrement bin. Furthermore, independent *t* test was used to investigate differences between the groups (young and old) for maximal torque during stimulated contraction, RR_T_, RR_MMG_ and MMG_0T_ (statistical significance *p* < 0.05).

In the graphs, the number of asterisks (*) indicates statistically significant differences as follows: *p* < 0.05 (*); *p* < 0.01 (**); *p* < 0.001 (***).

The data reported in this work come from signals detected during the tibialis anterior tetanic response used to compare the muscle mechanics at the onset of voluntary and stimulated contractions in young and old subjects in already published works (Cogliati et al. 2020).

## Results

**Fig. 3:**
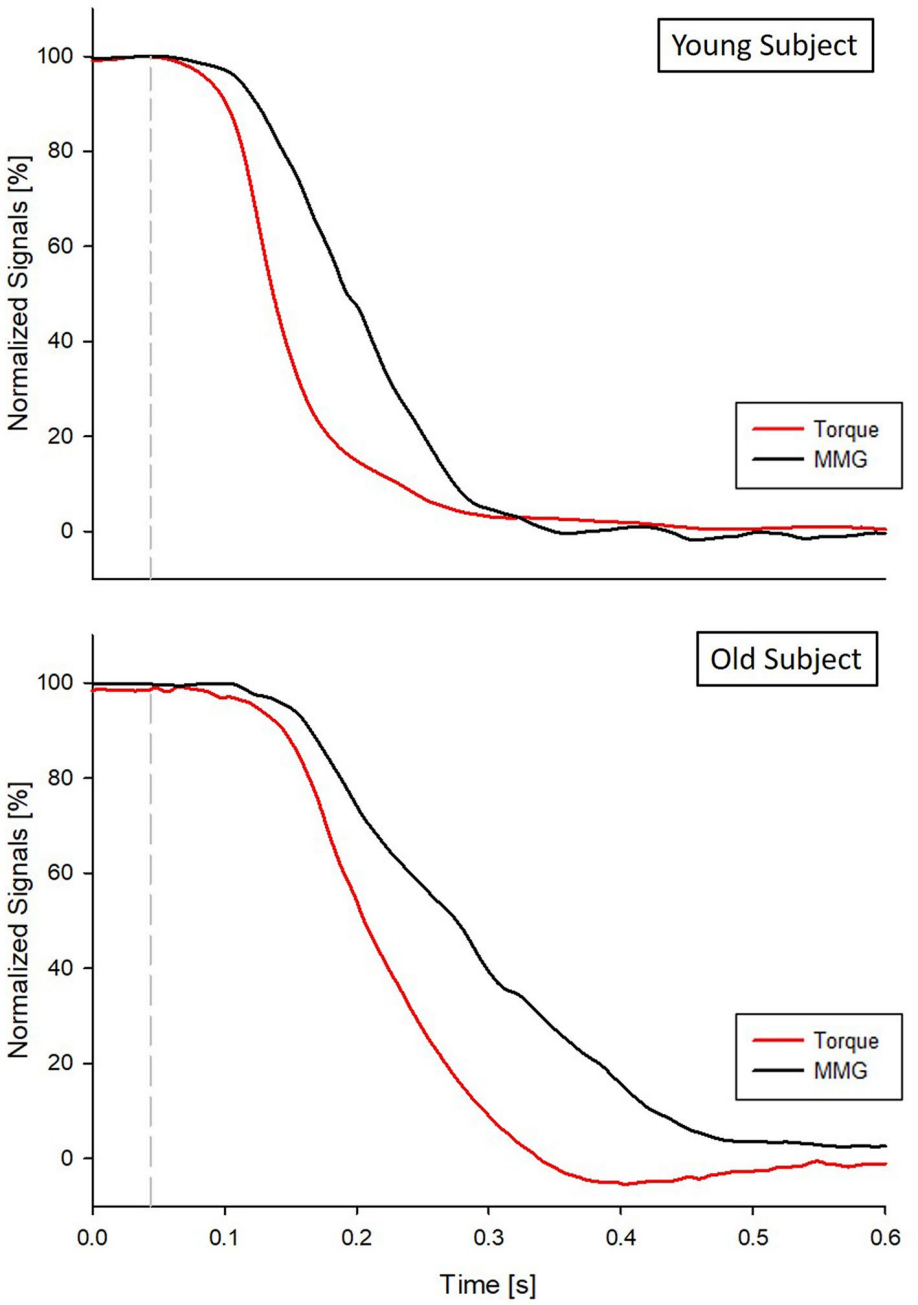
Normalized mechanical transients in two representative young (upper panel) and old (lower panel) subjects. The dashed gray lines mark the end of the electrical evoked activity. The influence of age on the beginning of the transient phase and its time behavior can be appreciated by comparison of the signals in the upper and lower panel

An example of the normalized torque (red line) and MMG (black line) signals from representative young and old subjects, from which the parameters listed in the previous section have been calculated, can be found in Fig. 3. The different time in the beginning of the two signals decay between young and old subjects as well as the different slopes of the two signals is evident through the relaxation process.

### Maximal stimulated contraction

The maximal torque in stimulated contraction was significantly different between young and old adults (4.9 ± 2.5 Nm for young and 2.6 ± 1.7 Nm for older; *p* < 0.001). A *t* test revealed a significant difference (*p* = 0.002) between young (3.01 ± 1.17 mm) and old (2.01 ± 0.73 mm) subjects for the maximal surface displacement transduced as MMG.

### Relaxation electromechanical delay (D_T_ and D_MMG_)

The two-way ANOVA revealed a significant effect of age (*p* < 0.001) and signal (*p* = 0.009) on D, but without an interaction between these factors (*p* = 0.354). Specifically, the older subjects had a longer delay compared to younger subjects. Moreover, the beginning of relaxation for the MMG started after the torque signals.

**Fig. 4:**
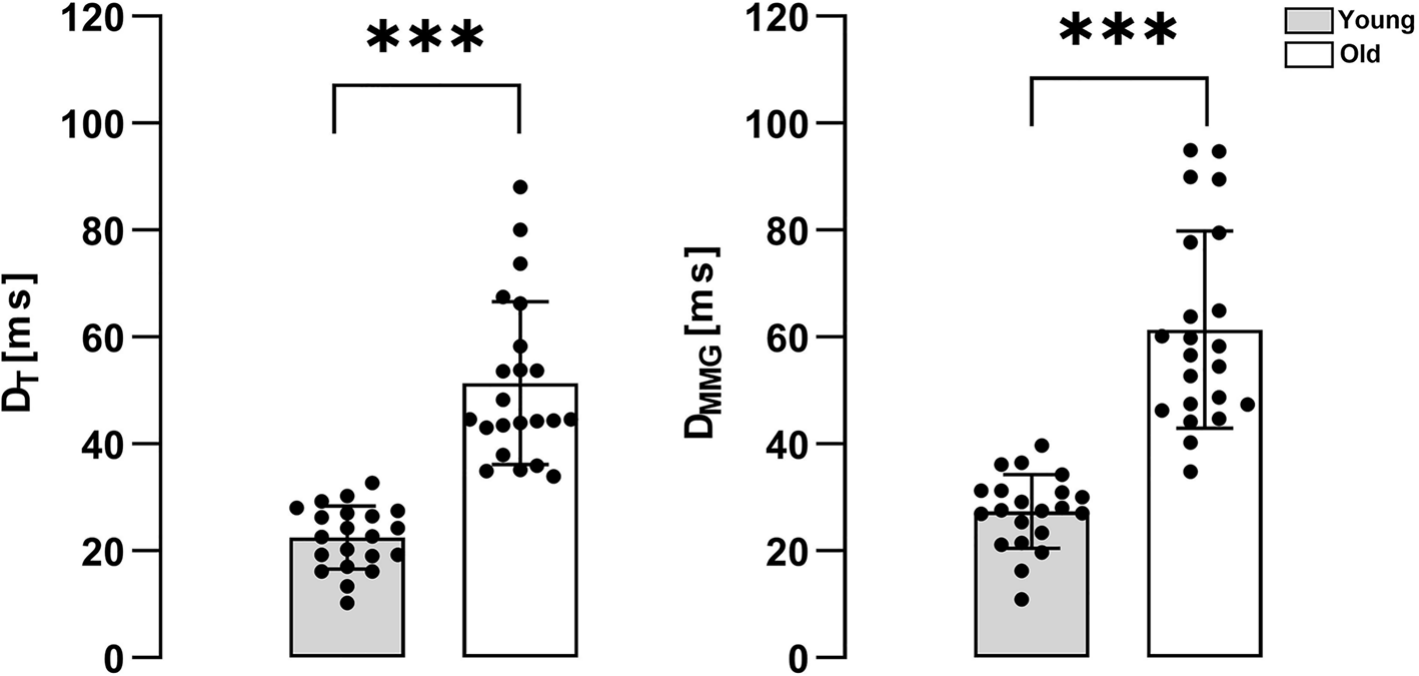
Delay on torque (left panel) and MMG (right panel) in the two groups. The differences in D_T_ and D_MMG_ are statistically different (*p* < 0.001) between the young (gray bar plot) and old (white bar plot) subjects

Torque. At the beginning of the relaxation phase, D_T_ was significantly different between young and older individuals (22.51 ± 5.92 ms for young and 51.35 ± 15.21 ms for older; *p* < 0.001) (Fig. 4).

MMG. D_MMG_ showed the same behavior as D_T_, with a significant difference being observed between young and older individuals (27.38 ± 6.93 ms for young and 61.41 ± 18.42 ms for older; *p* < 0.001) (Fig. 4).

### Rate of torque reduction and rate of MMG reduction (RR_T_ and RR_MMG_)

**Fig. 5:**
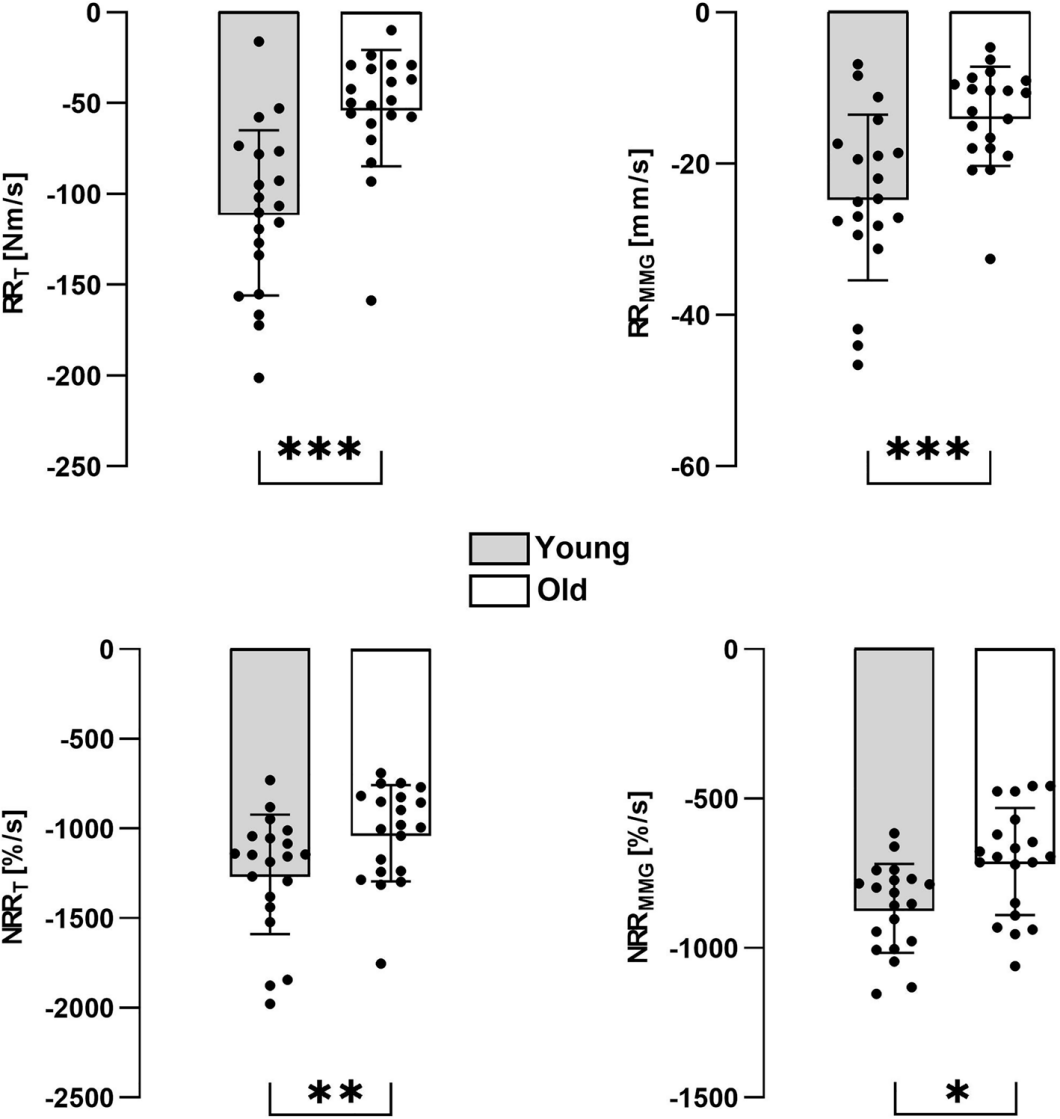
Rate of torque (left panel) and MMG (right panel) reduction in the two groups for the absolute (upper panel) and normalized (lower panel) signals. The differences in the values of the calculated RR are statistically different when Y and O data are compared (RR_T_ and RR_MMG_ *p* < 0.001; NRR_T_ *p* < 0.01; NRR_MMG_ *p* < 0.05)

During the decay phase after the stimulated contraction withdrawal, the maximal RR_T_ in young and old was − 110.4 ± 45.56 Nm/s and − 52.72 ± 32.12 Nm/s, respectively, showing a statistical difference between groups (independent *t* test; *p* < 0.001). Accordingly, the maximal RR_MMG_ in young (− 24.47 ± 10.95 mm/s) was significantly higher than in old (− 13.76 ± 6.54 mm/s) subjects (independent *t* test, *p* < 0.001). When considering the normalized signals, the results were similar. The two-way ANOVA revealed a significant effect of age (*p* < 0.001) and signal (*p* < 0.001) on the NRR, but without an interaction between these factors (*p* = 0.508). Specifically, the NRR was higher for young subjects compared to old and the decrease of the MMG signal was slower than the torque signal (Fig. 5). Moreover, the maximal NRR_T_ in young and old was − 1256.16 ± 333.36%/s and − 1026.26 ± 267.76%/s, respectively, showing a statistical difference (*p* = 0.004). Similarly, NRR_MMG_ was statistically different between young (− 867.79%/s ± 148.6%/s) and older (− 710.35 ± 178.84%/s) subjects (*p* = 0.044).

### Time interval for 80–20% signal reduction (TR_T_ and TR_MMG_)

The Two-way ANOVA showed a significant effect of age (*p* < 0.001) and signal (*p* < 0.001) on TR, but without an interaction between these factors (*p* = 0.468).

TR was lower for young subjects compared to old and the decrease of the MMG signal was slower than that of the force signal. TR_T_ was significantly lower for young (62.7 ± 17.08 ms) than old (81.65 ± 22.36 ms) (*p* = 0.022). Similar results were observed for TR_MMG_ in which a statistical difference was observed between young (104.7 ± 28.9 ms) and older (132 ± 31.48 ms) subjects (*p* = 0.001).

### Muscle compliance (MC)

**Fig. 6:**
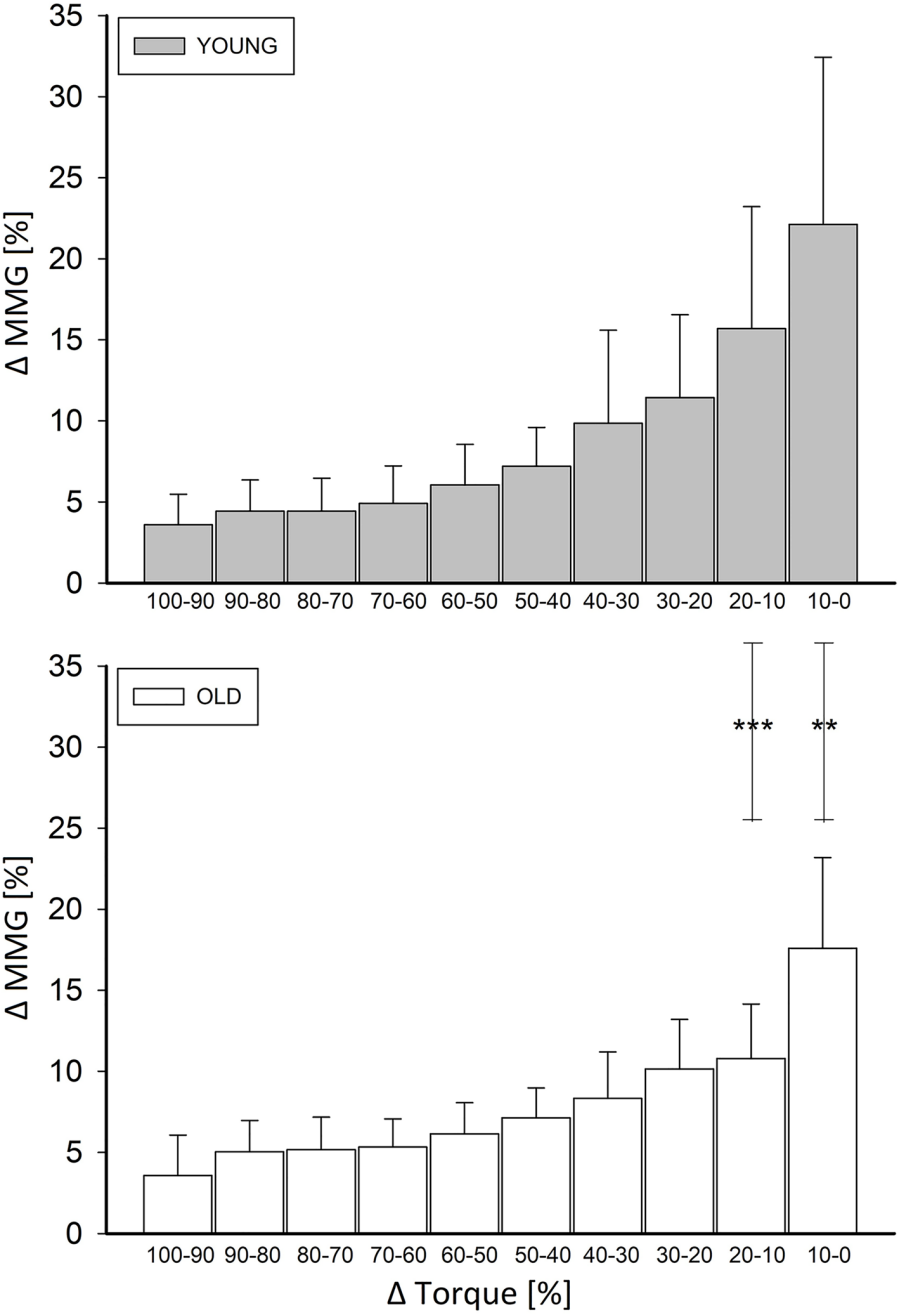
Muscle compliance to the re-elongation throughout the relaxation process. The amount of relative MMG variation for each of the ten bins of relative torque decrease (from 100 to 0%: 100–90%, 90–80%, 80–70%, …, 10–0%). Statistically significant differences between Y and O MMG variations can be found for the 20–10% (*p* < 0.001) and 10–0% (*p* = 0.001) bins

The two-way ANOVA revealed a significant effect of age (*p* = 0.016) and a significant effect of relative torque bin (*p* < 0.001) on the relative MMG changes. In addition, an interaction was found between these factors (*p* = 0.013). As shown in Fig. 6, within bin analysis indicated significant differences between groups for 20–10% bin (15.69 ± 7.5 [young] and 10.8 ± 3.3 [old], *p* < 0.001) and 10–0% bin (22.12 ± 10.3 [young] and 17.58 ± 5.6 [old], *p* = 0.001). The two groups did not show any other significant difference for the rest of the bins (*p* > 0.05).

### MMG at the end of torque reduction (MMG_0T_)

MMG_0T_ was significantly lower for young (5.41 ± 6.92%) than old (19.79 ± 11.95%) (independent *t* test, *p* < 0.001).

## Discussion

In this work, we assessed the torque and MMG reduction at the end of electrically stimulated contraction of the tibialis anterior in young and old subjects. Our main findings suggest that the age-dependent changes in muscle mechanics during the relaxation process with age are well described by the specific parameters obtained through the analysis of the dynamics of these two signals. These alterations could partially explain the specific features of alternating movements of walking in the elderly.

### Time course of torque and MMG

The comparative analysis of the time course of muscle length changes, indirectly measured using the MMG, and the force in human muscles has been already reported in the literature (Celichowski et al. 1998; Yoshitake et al. 2005, 2008; Orizio et al. 2008; Cogliati et al. 2020; Cè et al. 2017, 2013a, 2013c; Longo et al. 2017, 2016; Shinohara and Søgaard 2006; Jaskólska et al. 2003; Esposito et al. 2011, 2016). Only few papers, however, have investigated the phenomena during constant frequency tetanic stimulation to isolate the mechanical muscle response from the features of the neural control involved in voluntary contraction. In particular, several authors investigated the torque and MMG changes at the onset (Cè et al. 2017, 2013c, 2013a; Cogliati et al. 2020; Esposito et al. 2011, 2016) or at the end of evoked activity (Longo et al. 2016; Esposito et al. 2011, 2016; Cè et al. 2013c, 2014a, 2013b). The MMG detection techniques used the accelerometers (Longo et al. 2016; Esposito et al. 2011, 2016; Cè et al. 2013b, 2017) or the laser distance sensor (Cogliati et al. 2020; Orizio et al. 2013; Yoshitake et al. 2008). Here, the experimental design based on tetanic stimulation allowed, for the first time in humans, to describe the time relationship between the tension decrease and the muscle re-lengthening throughout the entire relaxation process. These results are in substantial agreement with the data reported in cats (Orizio et al. 2003).

We identified the end of electrical activity by analogy and mirroring the criteria generally accepted for the calculation of the time spent for electromechanical coupling during evoked contraction, in which the time interval between the first applied stimulus (onset of EMG signal) and the detectable tension increase is measured. As a consequence, the delay (i.e., the time interval spent before starting the relaxation process) was measured from the end of the electrical activity time instant and the beginning of torque and MMG decay (see Methods for its identification procedure). The comparison of our results with relaxation data from the literature is difficult given the scarce number of papers on this topic. Moreover, the delay of torque and MMG reported in some papers (Booth et al. 1997; Hespel et al. 2002; Cè et al. 2014a, 2013b; Esposito et al. 2011) were obtained using different techniques of signal detection (e.g., MMG was transduced by an accelerometer) and/or included the whole duration of the electrical activity evoked by the last stimulus of the tetanic train. On the contrary, in our procedure, this interval was not considered. For this reason, the absolute values of D_T_ and D_MMG_ reported are not directly comparable with those determined by the cited authors. Nonetheless, the literature data and ours agree that the D_MMG_ is always greater than D_T_. These differences, in the signals’ decay onset, as well as in the absolute (RR_T_ and RR_MMG_) and relative (NRR_T_ and NRR_MMG_) decay rates consistently indicate that force declines before muscle re-lengthening. This last complex process is influenced by several determinants such as the tendon shortening after the fall of tension and other possible factors (elastic energy storage restitution, changes in fluid distribution, variation in intramuscular pressure, etc.) as discussed by Orizio et al. (2003) and partly described by the model suggested by Uchiyama and Hashimoto (2011).

### Influence of age on muscle relaxation process monitored by torque and MMG

As mentioned above, D_T_ and D_MMG_ measured in this study are not directly comparable with those determined in several studies on the mechanical process of relaxation (Cè et al. 2013b, 2014a; Esposito et al. 2016; Longo et al. 2016, 2017) when the young population is considered. On the contrary, TR_T_ (reflecting the time spent from 80 to 20% force decay) is in line with those reported for the investigated subjects below 30 years old when the different muscle mass and the interval along the tension reduction period are taken into account (Cè et al. 2014a; Booth et al. 1997; Hespel et al. 2002). No data are reported in the literature about the muscle surface dynamics (MMG) during the relaxation phase after tetanic stimulation.

When the aged population is considered, no data about all the above considered mechanical parameters after tetanic stimulation can be found in the literature.

Our data indicate that in both torque and MMG, the beginning of decay and their reduction velocity (absolute and relative) occur later and are slower in old than young, respectively. To discuss this influence of age on the relaxation process, some basic consideration on its cellular mechanism can be useful. However, we have to keep in mind that the data retrieved from single fibers or isolated myofibrils cannot be directly used to explain experimental data obtained from the whole muscle tendon unit in humans. The cellular data can only highlight some factors that cannot be disregarded, but their action can be “filtered” by the much more complex situation of the “in vivo” experimental setup.

Muscle contraction is a consequence of the electromechanical coupling. Basically, it is determined by the increase of the cytosolic [Ca^++^] that removes the thin filament inhibitory state and allows the acto-myosin interaction or cross-bridge cycle. Vice versa, during muscle relaxation, the muscle active tension decreases as the decline of cytosolic [Ca^++^] restores the inhibitory state of the thin filament, so that myosins which end the cross-bridges cycle cannot start a new one. The sarcoendoplasmic reticulum calcium transport ATPase (SERCA) pump plays a key role given its function of removing calcium from the cytoplasm and moving it in the reticulum (Periasamy and Kalyanasundaram 2007). It is important to recall that when [Ca^++^] decreases below the threshold for thin filament activation, the number of attached active cross bridges initially slowly decays and then undergoes a fast collapse with a sudden increased rate of cross-bridge detachment (Cleworth and Edman 1972; Tesi et al. 2002). Thus, at the end of an isometric contraction, the tension decline takes place in two phases: in the first phase, the sarcomeres are kept in an isometric condition (slow phase), while in the following one the sarcomeres either shorten or elongate with a dramatic force fall (fast or chaotic phase) (for a review see Poggesi et al. 2005; Hill et al. 2021). Even if the extent at which the described phenomena can contribute to the time course of relaxation in intact mammalian muscle at physiological temperature (Hill et al. 2021) is not known, the body of knowledge on the muscular relaxation process must drive our interpretation on the differences in torque and MMG parameters in the young and old populations found in this study.

Following the interpretation outlined above, the rate of myoplasm Ca^++^ removal likely determines the time at which the threshold for tension decay is reached and probably is monitored by the D_T_ and D_MMG_ parameters. The difference in these parameters in the young and old may be due to differences in their SERCA characteristics. Indeed, Lamboley et al. (2014) reported that two different SERCA isoforms are expressed in fast and slow fibers. They appear to have different rates of calcium re-uptake. The slow fibers, with SERCA2 isoform, have a slower Ca^++^ re-uptake than the fast fibers with the SERCA1 pump. Given the prevalence of slow fibers in aged tibialis anterior (Orizio et al. 2016; von Haehling et al. 2010; Miljkovic et al. 2015), this can explain the longer delay of mechanical decay in old vs young. Moreover, Xu and Van Remmen (2021) reported a lower functionality of SERCA pump in aged subjects.

The steeper portion of torque and MMG decay, described by RR and TR, is likely and strongly influenced by the myosin cross-bridge kinetics. As shown by Belus et al. (2003) and Stehle et al. (2002), the chaotic and fast phase of tension decline is markedly dependent on myosin isoforms. Therefore, the above-mentioned prevalence of slow fibers in the tibialis anterior of elderly subjects can well explain the lower RR and NRR.

When the whole muscle tendon unit during relaxation after activity is considered, our data showed that the aged tibialis anterior re-elongates at a lesser extent for the same tension reduction compared to the young tibialis anterior (for muscle compliance in the 20–0% tension reduction). This is in agreement with the statistically significant greater value of MMG_0T_. The explanation can be found in the changes of muscle and tendon stiffness with aging. While tendon stiffness decreases (Magnusson et al. 2008), muscle stiffness increases with aging. The factors influencing the increase in muscle stiffness, passive resistance, in aged mammals are well described in Gajdosik et al. (2005) and can be summarized as: a. a substitution process of the contractile tissue with fat and connective tissue; b. a larger collagen amount in muscles dominated by slow twitch muscle fibers as those of old subjects. By analogy, the compliance of the muscle to re-elongation (tracked by MMG measure) due to the passive restitution of the elastic energy stored during tetanic stimulation may be influenced by the changes of the muscle viscoelastic properties with aging.

## Conclusion

Our data suggest that the degree of age-dependent impairment, during the different phases of gait cycle, can be partly related to the delay of the implied muscles to relax and re-elongate after their activity. This experimental setup measures the individual, internal muscle capacity to re-lengthening as part of the whole resistance to ankle joint angle changes when the calf muscles are activated. Eventually, our results contribute to the estimation of muscle mechanics properties’ alteration that may play a key role in the elongation of the gait phases in aged persons.

## Data Availability

Raw data are available upon request from the corresponding author.

## Abbreviations

D: Delay
MC: Muscle compliance
MMG: Mechanomyogram
MMG_0T_: MMG at the end of torque reduction
N: Normalized
RR: Rate of reduction
SERCA: Sarcoendoplasmic reticulum calcium transport ATPase
T: Torque
TR: Time of reduction
US: Ultrasound
